# The Core of Healthcare Efficiency: A Comprehensive Bibliometric Review on Frontier Analysis of Hospitals

**DOI:** 10.3390/healthcare10071316

**Published:** 2022-07-15

**Authors:** Thyago Celso Cavalcante Nepomuceno, Luca Piubello Orsini, Victor Diogho Heuer de Carvalho, Thiago Poleto, Chiara Leardini

**Affiliations:** 1 Núcleo de Tecnologia, Federal University of Pernambuco, Caruaru 55014-900, Brazil; 2 Dipartimento di Economia Aziendale, University of Verona, Via Cantarane, 24, 37129 Verona, Italy; luca.piubelloorsini@univr.it (L.P.O.); chiara.leardini@univr.it (C.L.); 3 Campus do Sertão, Federal University of Alagoas, Delmiro Gouveia 57480-000, Brazil; victor.carvalho@delmiro.ufal.br; 4 Departamento de Administração, Federal University of Pará, Belém 66075-110, Brazil; thiagopoleto@ufpa.br

**Keywords:** healthcare, hospitals, efficiency, productivity, frontier models, data envelopment analysis, stochastic frontier analysis, core publications, cluster analysis, bibliometrics, review

## Abstract

Parametric and non-parametric frontier applications are typical for measuring the efficiency and productivity of many healthcare units. Due to the current COVID-19 pandemic, hospital efficiency is the center of academic discussions and the most desired target for many public authorities under limited resources. Investigating the state of the art of such applications and methodologies in the healthcare sector, besides uncovering strategical managerial prospects, can expand the scientific knowledge on the fundamental differences among efficiency models, variables and applications, drag research attention to the most attractive and recurrent concepts, and broaden a discussion on the specific theoretical and empirical gaps still to be addressed in future research agendas. This work offers a systematic bibliometric review to explore this complex panorama. Hospital efficiency applications from 1996 to 2022 were investigated from the Web of Science base. We selected 65 from the 203 most prominent works based on the Core Publication methodology. We provide core and general classifications according to the clinical outcome, bibliographic coupling of concepts and keywords highlighting the most relevant perspectives and literature gaps, and a comprehensive discussion of the most attractive literature and insights for building a research agenda in the field.

## 1. Introduction

Hospitals are the largest cost factor in health care systems worldwide and are experiencing increasing pressure to improve the efficiency and quality of their services. Healthcare is one of the job sectors with the highest levels of complexity in business models, given the customers′ uniqueness. One way to better manage the nature of healthcare is through the incorporation of information technologies, medical innovations and new technologies, web platforms, data storage, analytical software, telecommunications systems and blockchain implementation to help deliver information and improve information security, speed of dissemination of information for administrators, professionals and decision-makers in healthcare organizations and improve the effectiveness of treatments [1,2]. According to Dwivedi et al. [3], the COVID-19 pandemic challenges the capabilities of health managers to make timely decisions to handle the pandemic efficiently and effectively.

At the same time, it forces the latter to rethink the essential elements of their operational processes in order to meet the new needs associated with the pandemic. Leite et al. [4] state that the COVID-19 pandemic has affected the operations of healthcare organizations in several aspects. These elements relate, for instance, to the refocusing of healthcare supply chain practices, the ability to maximize resource utilization and operations management practices. Understanding the impact of these factors allows health managers and policy makers to identify how to balance demand, resources and capacity properly. Therefore, according to Kamel and Mousa [5], hospitals can reappraise and restructure their existing plans to meet the organizational challenges brought by the pandemic through efficiency evaluation.

Hospitals consume several inputs (e.g., human resources, pharmaceuticals, equipment, etc.) to produce high-value outputs (e.g., outpatient visits, surgical operations, etc.). Hence, hospital efficiency analysis is about measuring the competence with which inputs are converted into valuable outputs. However, Kohl et al. [6] point out that estimating hospital efficiency is not straightforward. In this regard, one can emphasize that there are several methodologies to evaluate the efficiency of hospitals. According to Cordero et al. [7], efficiency scores of healthcare institutions can be estimated using either parametric or non-parametric methods. Both methodologies aim to assess production units and are based on productivity indicators, also known as technical efficiency (TE), that offer measures that characterize the operations of the units analyzed. Every econometric estimation of parametric functions has a definite mathematical form that is not very easy to identify [8]. Hence, the Stochastic Frontier (SF) is the most used parametric approach. It assumes that it is not possible to fully specify the function and allows for random noise. SF, therefore, takes into account the random component.

In relation to non-parametric methodologies, Data Envelopment Analysis (DEA) is the most widely used. DEA models allow the assessment of the relative efficiency of decision-making units (DMUs) by creating a production frontier using the best practice of the observed data [9]. It was developed by Charnes et al. [10]. In addition, it is used to rank and compare the efficiency of various entities. Thus, according to O’Neill et al. [11], DEA overcomes the weaknesses of parametric analysis because it does not require any assumption related to the functional form of the relationship between outputs and inputs. In addition, DEA can not only identify inefficient units but can also assess the degree of inefficiency. DEA uses linear programming to construct a piecewise convex linear segmented efficiency frontier, making it more flexible than econometric frontier analysis [12]. Moreover, DEA can include multiple inputs and outputs.

Despite these identified benefits, DEA presents some shortcomings. It attributes every deviation from the best practice frontier to inefficiency. Such deviations might be due to statistical noise. In addition, it is highly susceptible to the selection of variables and data errors. However, according to Kohl et al. [6], the most widely used tools for analyzing the efficiency of hospitals include DEA and other closely related tools. Furthermore, an important feature of the analysis of hospital efficiency through DEA concerns the fact that many authors (among others, Nayar and Ozcan [13]; Ferrier and Trivitt and [14], Wu et al. [15]) focused on the relationship between efficiency and quality. Indeed, according to Kohl et al. [6], the proportion of studies that include quality variables is likely to increase further and the inclusion of quality indicators should become the standard procedure in the future. However, it should be noted that the scientific community has not agreed on a common standard in dealing with hospital variables, and not all models are suitable to produce meaningful results from quality data processing.

Given the necessity to improve the performance of hospitals, this article aims to provide an overview of past, present and future research directions in the field of hospital efficiency assessment through DEA. More in detail, our study seeks to address the following collection of research questions (RQs):

*RQ1: Who are the most prominent contributions to the core literature of the DEA for the efficiency evaluation of hospitals?*
*RQ2: What are the main quantity and quality variables used in the literature on hospital efficiency evaluation through DEA?*
*RQ3: What are the main models and support approaches used in the literature on hospital efficiency evaluation through DEA? Which of these are most suitable for including different inputs and outputs combinations?*
*RQ4: What are the main gaps and overlaps in the literature on hospital efficiency evaluation through DEA? How can we use this perspective to construct and address future research agendas in the field?*

While previous research (e.g., Kohl et al. [6]) has attempted to analyze this topic through a literature study, we use bibliometric analysis [16,17] to reveal statistical patterns and provide an informative overview of key topical perspectives in the field of DEA application in hospital settings. Accordingly, this article not only sets out the most important issues in this field but also highlights those areas of research that have not been sufficiently developed, creating points of support for future research.

## 2. Systematic Review Design

On 13 January 2022, a systematic search was conducted at 10:48 a.m. (UTC+03:00) in the Web of Science™ database (https://www.webofscience.com/, accessed on 22 January 2022) [18]. Despite having less content than other bibliographic bases, the Web of Science (WoS) was chosen because it is recognized in the scientific community as the world’s most important repository for scientific literature. It contains more than 34 thousand journals and more than 171 million records in all fields of knowledge from 1900 to the present day on a global scale. Four query strings with three refinements were applied using the search tools provided by the website to retrieve the most relevant contributions in the field of healthcare efficiency. The results were extracted at 10:49 a.m., 10:53 a.m., 10:58 a.m., and 11:01 a.m. on the same day. At first, all searchable fields (such as topic, title, abstract, author keywords) in all collections (A&HCI, ESCI, CPCI-SSH, CPCI-S, SCI-EXPANDED, SSCI) were considered from 1980 (the first record) to 2022 using a straightforward query string Q1 “ALL = (healthcare AND efficiency)” resulting 15,594 records. Then refinements were made to those results. 

Table 1 reports all the query strings used in the systematic search design. The query links in this table directly address this review′s content on each specific instance. The second query string was applied to restrict the broad results, which included documents, such as bibliographies, corrections, database reviews, creative proses, meeting abstracts, and record reviews, among others, to articles only. The third query string was elaborated to meet research questions *RQ2*, *RQ3* and *RQ4*, restricting the results to parametric and non-parametric frontier applications only (DEA and SFA). The last string and refinement were elaborated to meet the research question *RQ1* restricting to hospital applications only.

**Figure 1:**
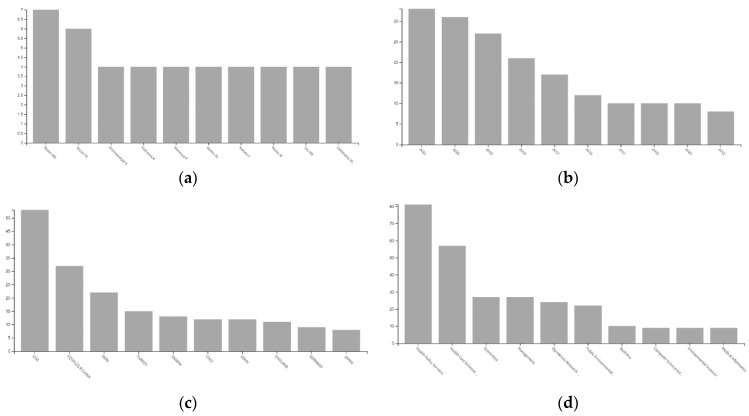
Results sorted by authors, year, country and field. Panels (**a**–**d**), illustrate the document distribution with bar charts per author (panel **a**), year (panel **b**), geographic region (panel **c**) and Web of Science categories (panel **d**).

Figure 1, panels a, b, c and d, illustrate the document distribution with bar charts per author (panel a), year (panel b), geographic region (panel c) and Web of Science categories (panel d). The most recurrent contributors are Michael D. Rosko (reported in the first and eighth bars of panel a) [23,24,25,26,27,28,29,30,31,32,33], Yasar A. Ozcan [34,35,36,37,38,39], Vivian G. Valdmanis [23,24,31,40,41,42], Ali Emrouznejad [43,44,45,46,47], Ryan L. Mutter [23,25,26,27,33], Kristina Kocisova [48,49,50,51], Paolo Mancuso [40,42,52,53], Yoshinori Nakata [54,55,56,57], Hongbing Tao [58,59,60,61], and Yuichi Watanabe [54,55,56,57]. The number of publications per year reported in the second panel (panel b) has a decreasing increase (increase with a decreasing rate), especially over the past six years. The higher growth in the number of publications is from 2014 to 2015, a 100% increase from five to 10 publications. There was a particular tendency for COVID-19-related publications over the past years in hospital applications. The mean growth rate is 0.1739 over the past decade (2020–2021: 0.0645, 2019–2020: 0.1481, 2018–2019: 0.2857, 2017–2018: 0.2352, 2016–2017: 0.4166, 2015–2016: 0.2000, 2014–2015: 1.0000, 2013–2014: −0.2857, 2012–2013: −0.1250, 2011–2012: −0.2000).

**Figure 2:**
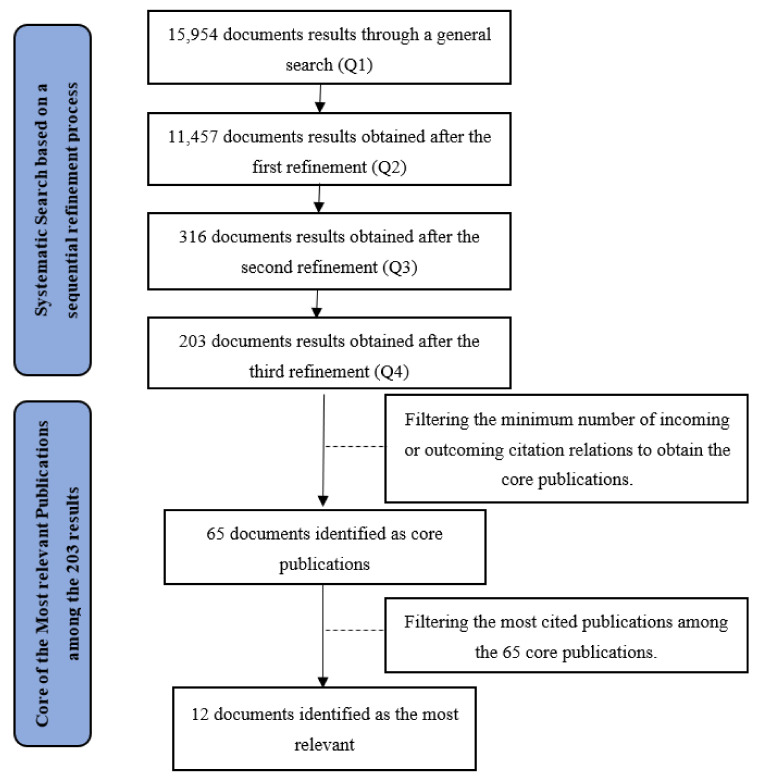
Flow Diagram with the different phases of the systematic review (according to Moher et al. [64] PRISMA scheme).

After the third refinement, we applied the Core Publication methodology [62,63] for selecting the most relevant records strictly related to the field of efficiency analysis in hospitals. Sixty-five papers out of the 15,954 documents from the general search (Q1) were identified as core publications in this exercise. After classifying, filtering and investigating this network, we retrieved the twelve documents identified as the most relevant based on citation scores. They are discussed in the following section. Figure 2 illustrates the Prisma Diagram for this systematic review application.

The Web of Science Systematic Search based on a sequential refinement procedure departed from 15,954 document results applying the general search query Q1. Then, 11,457 documents results were obtained after the first refinement applying the query string Q2, 316 documents results appeared after the second refinement applying the third query string Q3, and finally, 203 documents results were obtained after the third refinement applying the fourth query string Q4. From this point, we resort to the Core Publication Methodology of van Eck and Waltman [62], by filtering the minimum number of incoming or outcoming citation relations, resulting in 65 documents identified as core publications. Lastly, 12 documents were ranked as the most relevant based on their citation score. Figure 2 summarizes the systematic search.

## 3. Core Publications and Research Impact

The Core Publication Methodology is an interesting approach for selecting the core, i.e., the most important publications related to a specific field. Many networks of scientific literature may include publications that, despite their importance in terms of content quality or citations, are weak related or completely unrelated to the investigated field [65]. According to van Eck and Waltman [62,66], a Core Publication is a paper with (at least) a certain minimum number of interactions with other core publications. These interactions are counted in the number of incoming and outgoing citations. This methodology keeps in the core of a network of scientific literature only the relevant contributions to the investigated area, regardless of the van Eck and Waltman citation scores [62,66].

By definition, at least one core publication must be related to another core publication (by citing or being cited). Thus, the minimum number of core publications for any bibliometric network is two. Following Nepomuceno et al.’s [65] approach for selecting the most relevant and restricted network, we define four as the maximum–minimum threshold of citation links in which at least two core publications can be identified. This means that each core publication in this network has citation relations with at least four other core publications. Above this threshold, no core publications can be identified (i.e., there is no core publication with five incoming or outgoing citation relations with another core publication).

### 3.1. Classification

**Figure 3:**
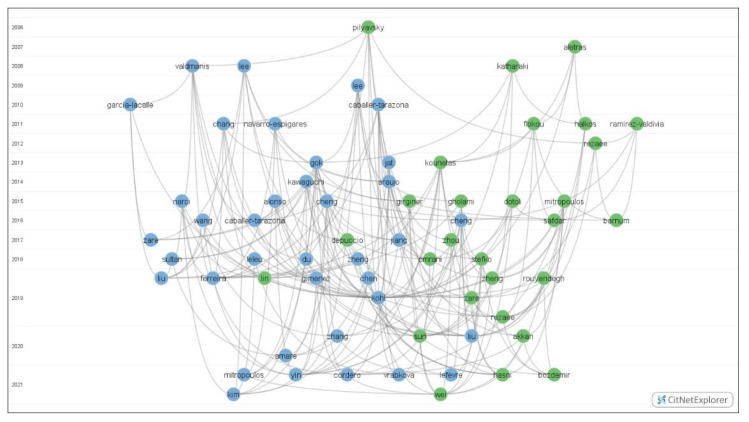
Timeline-based network visualization for the core publications.

After applying the maximum–minimum threshold of citation links, 65 documents were identified as core publications in the field of hospital efficiency analysis. Table 2 reports the core publications classified into two groups using the clustering technique from van Eck and Waltman [62,66]. The most common inputs, outputs, frontier models and methods are highlighted for each group. Figure 3 illustrates the timeline-based citation network of the 65 core publications represented in blue, mainly located on the right side of the visualization (left side of the reader) and green (mainly located on the left side of the visualization (right side of the reader).

The most commonly used inputs in more than 80% of the core publications are hospital beds (intensive care unit beds, emergency beds, psychiatric, chronic, tuberculosis and leprosy beds [47,74]), physicians (specialists and non-specialists), nurses and other medical and non-medical staff (such as ancillary services personnel, pharmacists, laboratory and other technicians, administrative personnel, radiological technologists, midwives, and dietitians [43,47]). They are often measured in Full Time Equivalent (FTE) [1,41,52,60,67]. Another common but less recurrent input is expenditures. Salaries and other financial or administrative expenditures, such as interest costs [72], spending on medicines and on purchase of goods and services [73], rural medical and general healthcare expenditures [74] are often reported in local currency. Municipal subsidies to cover deficits are also reported in the work of Kawaguchi, Tone and Tsutsui [72] as an input related to healthcare expenditures.

Instead of the total number of beds (aggregate or by medical specialties), some works employ the number of available beds or “the actual number of open beds” [60,85,88], which according to the authors, represent a better proxy for capital inputs in the healthcare production system. The hospital area, medical business area, the number of doctor’s offices and surgical rooms are examples of physical inputs related to the building where the healthcare services are provided in the works of Araújo [77] and Liu [88]. The works of Tao Du [81] and Tao Liu et al. [74] are macro-efficiency evaluations rather than hospital institutional assessments using the number of healthcare institutions in 31 provinces of China as input. Lastly, two unusual and interesting inputs are the service complexity reported in the work of Lee, Yang and Choi [71], defined as the total number of diagnostic and special services provided by the hospital, and the unmet medical needs reported in the work of Mitropoulos [89] exhibiting the population seeking healthcare but which cannot have access to it due to financial and service barriers in the healthcare system.

The outputs are more diversified than inputs in the applications. The most common healthcare products regarded in the hospital efficiency core applications are the number of discharges [7,70,71,75,83], the number of surgeries [67,77,78,79,83,86], and the total number of inpatient and outpatient treatments, diagnoses and healthcare income [68,77,81]. Childbirth-related outputs are antenatal checkups, deliveries (such as cesarean-section deliveries), abortions, post-natal care, and the number of male and female sterilizations [83,84,86]. Two interesting outputs related to hospital bed management are bed utilization and turnover rate [81]. Case-mix adjusted healthcare admissions and discharges [70,71,75,78,79], mostly based on diagnosis-related group (DRG) systems, are another interesting output integration used to consider the complexity of diseases and clinical specialties. The complexity and differences among medical specialties impact any efficiency analysis based on quantitative outputs, making it easier or more difficult to expand that related product [117]. This is a relevant topic for consideration in this field. 

Efficiency analyses of hospitals are filled with applications containing undesirable outputs composing healthcare units’ production technology, which aims to be reduced instead of expanded in the DEA linear formulation. Examples are in-hospital mortality, inpatient days (length of hospitalization stays) and readmissions [65,66,83,85,86]. Other outputs present in Network DEA models are known as links (intermediate products), i.e., an output that is at the same time an input for another department of the service unit. This is the case of Kawaguchi et al. [72] Dynamic-Network Data Envelopment Analysis model which uses “number of beds” as a link variable from Division 1 (administration) to Division 2 (medical examination) in the efficiency estimation of Japanese hospitals. Other studies, such as Amare et al. [76] and Du [81] are two-stage approaches used to evaluate the impact of quality environment variables or exogenous (independent) factors, such as the CEO’s administrative service years, educational status, the distance of the CEO’s residence from the facility, incentive packages for the employee, patient waiting time, among others. Another interesting work

Some controversial outputs found in the literature implicate using exogenously determined variables that are not sufficiently under the control of healthcare units and limit the scope of the production possibilities of hospitals. Exogenous outputs, such as outpatient visits, ambulatory and emergency room visits, number of ordinary admissions, ICU inpatients, emergency inpatients, first consultations and successive consultations [67,68,70,71,75,78,79,84,85,86] are some examples. They depend on social, demographic, spatial and economic covariates that affect people’s health conditions that hospitals do not control to expand. It is possible that, in a comparative perspective, efficient hospitals produce many consultations or admissions compared to others with similar production structures because they are faced with a bigger population (demographic factor) or poorer, less-educated regions (socioeconomic factor). When control variables are not included in such analysis, results might bias the distribution of inefficiencies. According to Nepomuceno et al. [118], such analysis can jeopardize the objective measure of technical efficiency and the inclusion of exogenously determined outputs as non-discretionary inputs are suggested.

### 3.2. Discussion on the Most Relevant Contribution

**Figure 4:**
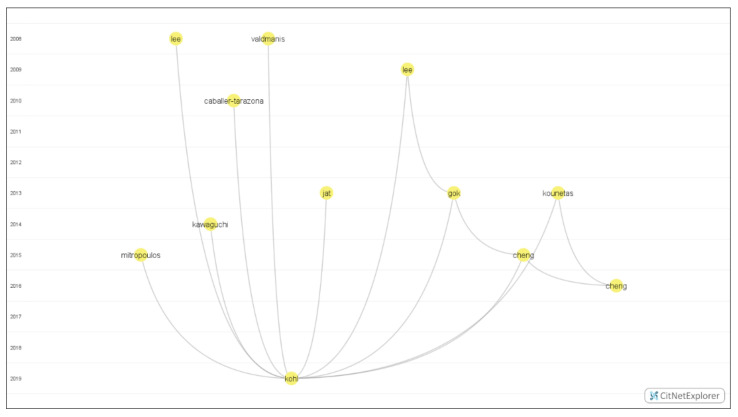
Timeline-based network visualization for the most relevant contributions.

This subsection is dedicated to a comprehensive discussion on the most relevant contributions based on the attractiveness metric of the 12 top-cited core publications. The attractiveness of a publication is defined as the number of Google Scholar citations divided by the publication period. This bibliometric measure provides a fairer comparison between old and new publications by offering a number of citations per unit of time. For instance, the work of Valdmanis, Rosko and Mutter (2008) [23] has the second-highest number of Google Scholar citations (until January 2022) with 152 citations, but it is the sixth-ranked core publication with about 0.96 citations per month (paper published on 20 September 2008) compared to the work of Gok and Sezen (2013) [83] with fewer citations (136 citations) but published more recently (on August 2013), reporting about 1.35 citations per month. Figure 4 and Table 3 report the most relevant core contributions in the efficiency analysis of hospitals.

Kohl et al. (2019) [6] developed a literature review covering 262 papers with DEA applications in hospitals to discover gaps not covered in a timeline from 2005 to 2016. Their literature search was performed on Google Scholar, Science Direct, and PubMed, including works only in English, detecting an increasing trend in the number of articles published along the defined timeline, with the peak occurring in 2016. In terms of the geographical distribution of the publications, it was detected that most studies came from Europe (with 96), followed by Asia (66), in the second rank, and North America (64), in the third rank, and these three regions presented a tendency in increasing the number of papers. Africa (with 21) appeared in the fourth rank and was the only region presenting a decline. About the types of research questions, the authors detected the following distribution: 100 publications related to “specific management questions”, 99 related to “performing DEA”, 48 related to questions on “new applications or methodologies, and 36 related to the “effects of reforms”. There was a tendency to increase the number of papers in the timeline for each question cluster. In terms of model specification, it was noted a remarkable trend for the use of quality parameters, and related to the DEA model selection, two basic models–CCR (Charnes, Cooper, and Rhodes model) and BCC (Banker, Charnes, and Cooper and model)–appeared with most of the applications (112 and 144, respectively).

Gok and Sezen (2013) [83] analyzed the effects of efficiency and structural quality of Turkish hospitals on patient satisfaction. They applied DEA using data from 524 hospitals from which a sample of 348 observations was extracted, and multiple regression analysis was applied to evaluate the relationship between patient satisfaction, structural quality, hospital efficiency, and institutional factors. The study detected that small-size hospitals are relatively more efficient, having higher patient satisfaction. In contrast, large hospitals provide comparatively higher quality care, tending to increase their infrastructure to meet the care process more. Structural quality proved to significantly impact patient satisfaction, with efficiency negatively moderating the relationship between structural quality and satisfaction.

The study of Cheng et al. (2015) [61] examined the technical efficiency and productivity of Henan county hospitals during China’s Healthcare Reform Plan started in 2009, using two methodologies (Stochastic Frontier Analysis–SFA, and DEA) seeking to determine whether and how efficiency is affected by a series of environmental and institutional factors. The study detected a considerable space for technology efficiency improvement, with 98.2% of the sample, with 114 hospitals considered inefficient in 2010 and 2011 and 91.2% in 2012.

Kawaguchi, Tone and Tsutsui (2014) [72] developed the first study applying the dynamic-network (DN) DEA model in healthcare, performing an evaluation of the policy effect of Japan’s municipal hospitals’ reform, focusing on efficiency improvements both within the hospital and within two separate internal hospitals organizations. They selected a hospital with more than 300 beds among 1000 hospitals. The results obtained implied three policy implications: (i) the dynamic change in period-divisional efficiency scores between 2007 and 2009 was relatively small in administrative and medical divisions, not finding positive policy effects on average on Japanese municipal hospitals; (ii) looking for individual hospitals, the study could not show a strong correlation between the efficiency scores of administration and medical divisions; (iii) focusing on the efficiency improvements of two divisions in individual hospitals, the ratio of hospitals where both divisions improved efficiency was 16.96% while the ration of hospitals where efficiencies were maintained or reduced was 58.92%, and the rate of hospitals where change direction differed between both divisions was 24.12%. The authors conclude they did not find any significant efficiency improvement even with the implemented municipal hospital reform policy.

Kounetas and Papathana-ssopoulos (2013) [107] developed a study intending to measure Greek hospitals’ performance using SFA and DEA, using a dataset consisting of 114 hospitals from 130 in a statistical record from the Greek Ministry of Health and Social Solidarity Welfare for 2008. The authors defined the hospitals’ performances as not encouraging, demonstrating that their study’s technical efficiency scores are lower than in previous ones. They also found that bed occupancy ratio was inversely related to technical and scale inefficiency and that the type of hospital improved technical efficiency while scale inefficiency is unaffected.

Lee, Yang and Choi (2009) [71] assessed the relationship between hospital ownership and technical efficiency in a managed care environment. The data used in the study are related to 435 hospitals in Florida (United States) from 2001 to 2004. DEA was used to calculate hospital technical efficiency scores, determining that non-profit hospitals were generally more efficient than for-profit ones in the four-years timeline explored. No difference was found between non-profit and for-profit hospitals with less than 100 beds and between 250 and 399 beds. However, a statistically significant difference was detected between non-profit and for-profit hospitals with 100 and 249 beds and those with more than 400 beds.

The study by Mitropoulos, Talias and Mitropoulos [109] is dedicated to accounting for the impact of statistical noise in DEA. They proposed a combination of a chance-constrained DEA (CCDEA) with a Bayesian approach, making an empirical application in 117 Greek public hospitals, divided into 17 primary hospitals, 71 secondary, and 29 tertiary. The DEA input variables used were: the number of doctors, number of other personnel, number of beds, and total operating cost. The outputs defined selected were: the annual number of inpatient admissions and aggregated scheduled and emergency outpatient visits. The authors’ findings confirmed the efficacy of the Bayesian-CCDEA model, showing that it can also be applied in contexts where there is uncertainty to provide comparisons between technical and technology gaps among different groups.

Valdmanis, Rosko and Mutter [23] applied congestion analysis to assess trade-offs between quality and efficiency in 1377 urban hospitals in 34 states in the United States. This analysis is a variation of DEA, and for this study, the authors considered as inputs: the number of bassinets, the number of licensed and staffed beds minus the number of beds in nonacute units; licensed and staffed “other” beds, the full-time equivalent number of registered nurses, number of licensed practical nurses, number of medical residents and number of other personnel. As the outputs, the authors defined: the number of Medicare case mix index adjusted admissions, total surgeries, total outpatients’ visits, total births, and total other patient days. Among their findings, they detected some characteristics associated with inefficiency and quality congestion in the hospital studied: 3% of the total inefficiency can be attributed to quality congestion; most hospitals in the sample were operating at diseconomies of scale, suggesting that slack, mainly on beds, can be an inefficiency related factor; in teaching hospitals, the main source of inefficiency was diseconomy of scale; high-technology equipment was associated with higher quality.

Cheng et al. (2016) [60] studied the efficiency and productivity changes in 48 township hospitals in the three-tier rural healthcare system of China, from 2008 to 2014. They applied the bootstrapping DEA to estimate technical efficiency, pure technical efficiency, and scale efficiency and then they applied the bootstrapping Malmquist productivity index to calculate productivity changes within the studied period. Their findings showed that the average technical and pure technical efficiency of the hospitals in the sample was relatively low, indicating a great potential for improvement. In contrast, the average scale efficiency was relatively high; most inefficient hospitals were in decreasing returns to scale, and there is a considerable possibility of improvement for the output if this case is better managed.

The study by Caballer-Tarazona et al. [79] was focused on the efficiency of 22 hospitals in the Valencian Community (Spain). In this study, the authors designed and applied a healthcare performance evaluation system based on DEA to discover and improve potential inefficiencies. They used the number of doctors and beds as input variables and the number of admissions weighted by the case mix, first and successive consultations, and surgical innervations as output variables. They highlighted some results regarding the different analyses to approach the efficiency measuring: the DEA model was considered more useful when studying each service separately instead of studying the overall efficiency of a given hospital; the DEA model can be difficult to be used by hospitals’ managers, and it implied in the construction of user-friendly indicators to support efficiency measuring (namely the indicators “Weighted admissions/doctors” and “Interventions/doctors”), as an alternative to DEA.

Jat and Sebastian [84] developed a study to evaluate the technical efficiency of 40 public hospitals’ maternal healthcare services in Madhya Pradesh (India). They applied DEA, collecting data from the hospital from January to December 2010, using the health management information system and other records from the state’s health management agency. The authors defined their study as the first attempt to achieve the defined objective using DEA, describing that the average pure technical efficiency of 0.90 can produce the same number of outputs by saving 10% of the inputs, implying that the input savings can be applied to provide more healthcare services for more people through the community health centers in rural poor areas. Another result highlighted by the authors was that 50% of district hospitals are operating at a less-than-optimal level, and eleven of these hospitals obtained scores below 0.8, implying that the inefficient hospitals could significantly improve their efficiency with better resource management.

Lee, Chun and Lee [70] explained the relationship between the case-mix specialization index and the efficiency of inpatient hospital care services using data from 106 acute care hospitals in Seoul (South Korea). For this purpose, the authors applied DEA with the collected data using the case-mix specialization index (in the study, they calculated it as the Information Theory index) to measure the extent of hospital specialization. The results obtained indicated that only 10.3% of the 106 hospitals were relatively efficient in the market, providing practical implications for hospital managers to identify areas for improvement based on the examined variables in the inefficient hospital.

## 4. Cluster Analysis

**Figure 5:**
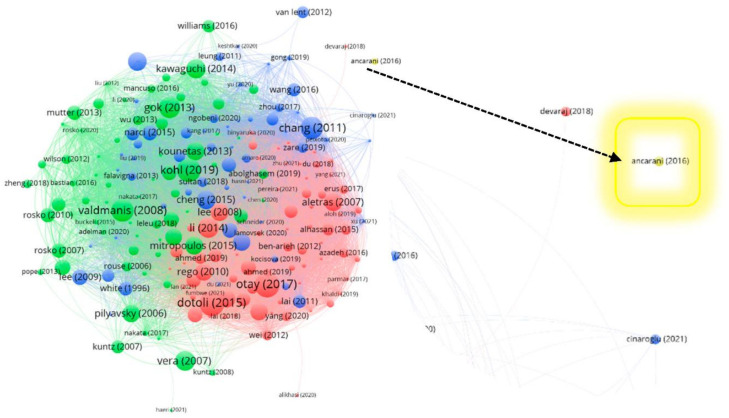
Distance-based network visualization for 203 Web of Science papers.

This section is dedicated to a general classification and discussion of the bibliographic network containing the 203 references on hospital efficiency retrieved after the third refinement (Q3). Figure 5 illustrates the distance-based bibliometric network using the Vosviewer tool [62,66]. This network was constructed on the bibliographic coupling of works. Links are created when two publications cite the same third publication [119]. The visualization parameters are attraction = 8, repulsion = 0, resolution = 1 and the minimum cluster size = 58. Based on the bibliographic coupling network, we construct three co-occurrence landscapes and three radar charts for discussing a research agenda and highlight the most prominent perspectives on healthcare efficiency.

The relations among publications are represented by the number of links and the distance of nodes. For instance, the work of Cheng et al. (2015) [61] on evaluating the technical efficiency of Chinese county hospitals (blue node in the center of this visualization) is more related to the work of Sultan et al. (2018) [69] on measuring the efficiency of Palestinian public hospitals (smaller blue node just above the work of Cheng et al. 2015) than it is to the work of Van Lent et al. (2012) [120] on exploring improvements in patient logistics in Dutch hospitals (blue node on the extreme upper-right side of the visualization). The colors red, green and blue (and one small yellow cluster composed of only one publication, Ancarani et al. (2016) [52]) indicate the groups to which a publication about hospital efficiency belongs. They are used to classify the network in the following subsection.

### 4.1. General Classification

Table 4 reports the general literature classification into the four clusters. The red cluster has 20 core publications out of 73 publications [28,42,46,50,55,61,64,73,75,76,77,78,82,86,87,89,91,94,95,96]. It is related to mostly econometric approaches to aid the efficiency analysis, such as Tobit Regressions, Bootstrap and Principal Component Analysis, and Multi-criteria and decision support approaches, such as AHP, Fuzzy Cognitive Map, Stochastic Multicriteria Acceptability Analysis and Multiple Stage approaches. This cluster is dedicated to the efficiency analysis of healthcare units involving environmental and socioeconomic contexts.

The green cluster with one publication less than the red one is the second biggest group of publications. It has 20 core publications out of 72 publications [1,2,26,45,49,51,52,54,56,57,58,59,62,65,68,71,72,74,83,85]. This cluster includes financial or managerial perspectives in the performance management of hospitals. Developments on the recurrent trade-off between healthcare quality and technical efficiency, patient satisfaction, benchmarking, uncertainty, and health payment systems are reported in many publications present in this group. This cluster also includes information, communication and decision-support technologies, such as picture archiving and topics about healthcare information technology performance, electronic medical record and discussions about endogeneity and appropriate incentive structures.

The blue cluster has 23 core publications out of 58 publications [16,29,31,34,41,43,44,47,48,53,60,63,66,67,69,70,79,80,84,88,90,92,93]. This cluster is about interesting frontier applications in hospital sectors and healthcare categories that are not usual in the field of hospital efficiency analysis. Some of the related applications are in sectors and healthcare services related to cardiovascular diseases, acute care, pharmacies, rural medical systems, laboratories, surgery, cancer screening, and pet cancer. The last (yellow) cluster refers to only one publication (Ancarani et al. [52]) about different religious perspectives and how they might impact the efficiency of hospitals.

### 4.2. Co-Occurrence Networks

**Figure 6:**
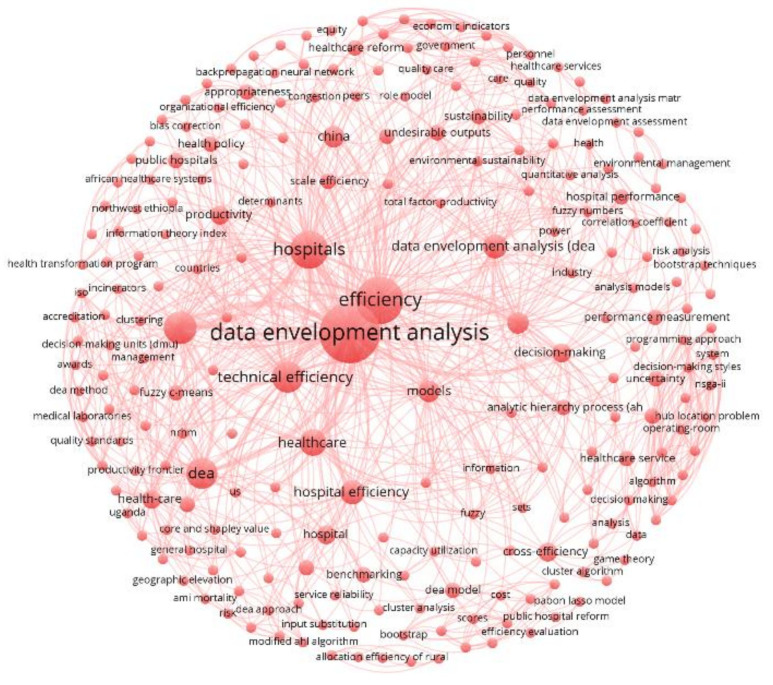
Keywords Co-occurrence for the Red Cluster.

**Figure 7:**
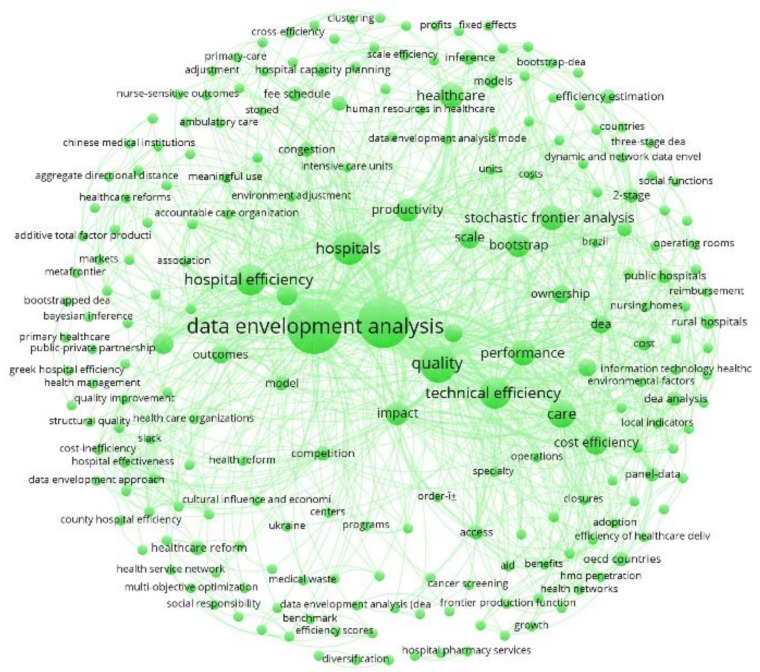
Keywords Co-occurrence for the Green Cluster.

**Figure 8:**
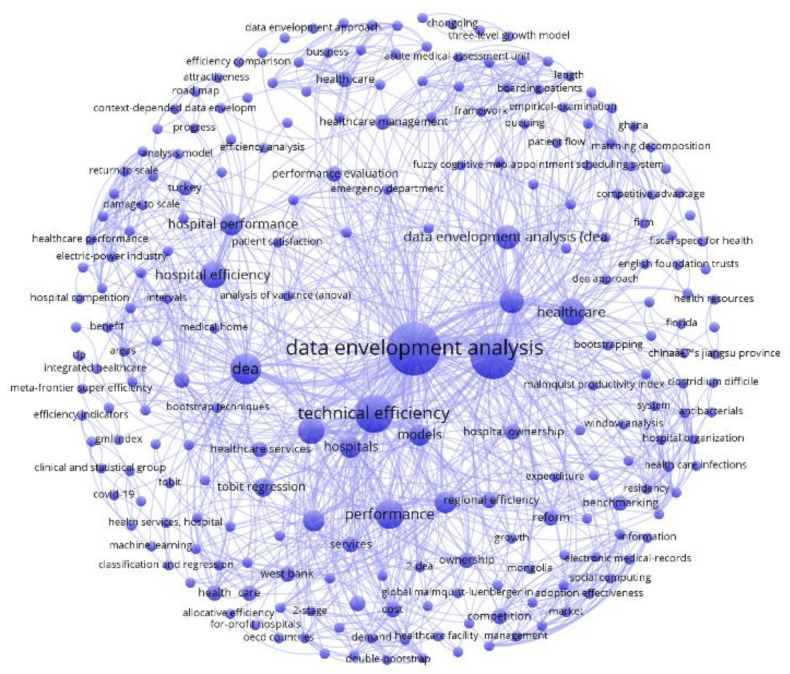
Keywords Co-occurrence for the Blue Cluster.

Each cluster is analyzed and separated for the purpose of this investigation. The bibliographic information for each documentation was treated using Mendeley Desktop version 1.19.3. The co-occurrence networks in Figure 6, Figure 7 and Figure 8 are the products of such bibliographic treatment exported into a .ris file. Keywords in any systematic review are essential sources for knowledge abstraction because they summarize in one or a few words the content of a document and main concepts of great significance in the field of scientific investigation. Consider *i* = 1, 2, 3, …, *n* keywords to be mapped in each publication cluster. For any *j* ≠ *i* keywords, the number of co-occurrences of the two keywords *j* and *i* is the number of publications in which both keywords occur together, denoted by $C_{i,j}$ = $C_{j,i}$, and the total number of co-occurrences for the keyword *i* is $C_{i}=\underset{i\neq j}{\sum }C_{i,j}$ [62,228].

Full counting was applied as the citation counting method, which is the most common approach where each co-occurrence link has the same weight [62,228]. The total number of keywords for the red cluster is 232 (from 2 to 156 total link strength). This number for the green and blue clusters is 221 (from 2 to 143 total link strength) and 229 (from 2 to 175 total link strength). Table 5 reports the most recurrent keywords (with the minimum number of three occurrences for a keyword) for each cluster (see the keywords co-occurrence map visualizations in Figure 6, Figure 7 and Figure 8).

### 4.3. Outlining a Research Agenda

One of the most significant challenges to developing innovative ideas and models to cope with problems of a quantitative nature is that we can determine all the potential determinants affecting the object of study. Most of the time, we can only measure the impact of some known potential determinants of the investigation, i.e., testing whether the true values of variables are close to following a predictive path designed by the analyst. This is also a challenge in identifying relevant research topics to build a research agenda that comprehends the social needs for scientific advances. An interesting avenue to pursue this purpose is reported in Daraio et al. [231], which use a similar bibliometric methodology to assess the relevance of different applications in the field of efficiency analysis. 

The co-occurrence of the concepts, perspectives, approaches, methods, categories, activities, and areas can be used to define a degree of generality that identifies gaps and overlaps in the research field. The Degree of Generality (DG) is defined as the inverse of the relevance score [231]. This exercise can stimulate knowledge discovery by identifying “context clues” left on titles, keywords and abstracts for interesting topics that can be explored in future research agendas on the field of investigation. Table 6 reports the main topics extracted from each cluster network classified per area with the range of generality for directing a research agenda in the field of healthcare efficiency. According to Daraio et al. [231], degrees of generality between 0 and 1 are reserved for limited topics (terms) and fields of application, neither sufficiently covered by scientific publications nor co-occurring with other terms or fields.

All the topics reported in Table 6 have a DG equal or below 1, with the exceptions on multiple stage approach (DG = 1.035), undesirable output (DG = 1.438), uncertainty (DG = 1.534), public hospital (DG = 2.027) and complexity (2.178) in the Red Cluster, and for the Green Cluster the topics effectiveness (DG = 1.460) and readmission (DG = 1.810). The most prominent topics to be prioritized for promoting a research agenda according to the bibliometric network are benchmarking (0.624), integrated quality (0.333), and fee schedule (0.250) with regard to Financial and Managerial Perspectives; allocation efficiency (0.250), directional distance functions (0.635) to principal component analysis (0.458) about the methods and support approaches.

**Figure 9:**
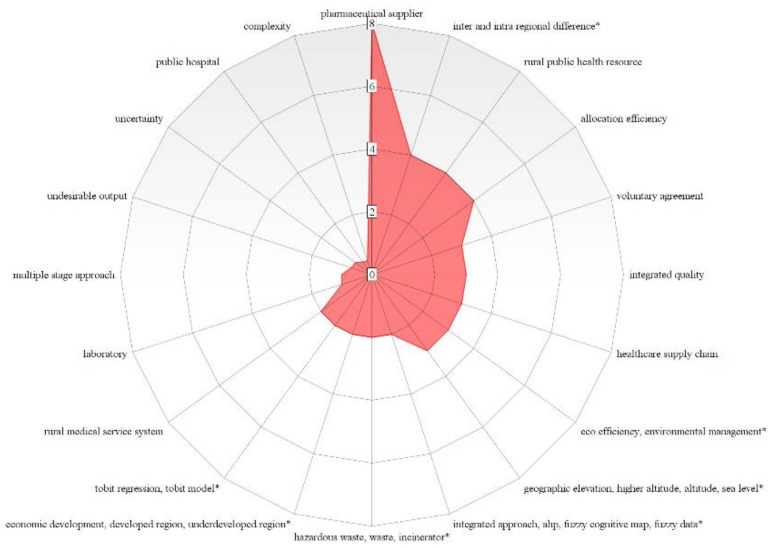
Hot Topics for a Research Agenda in the Red Cluster.

**Figure 10:**
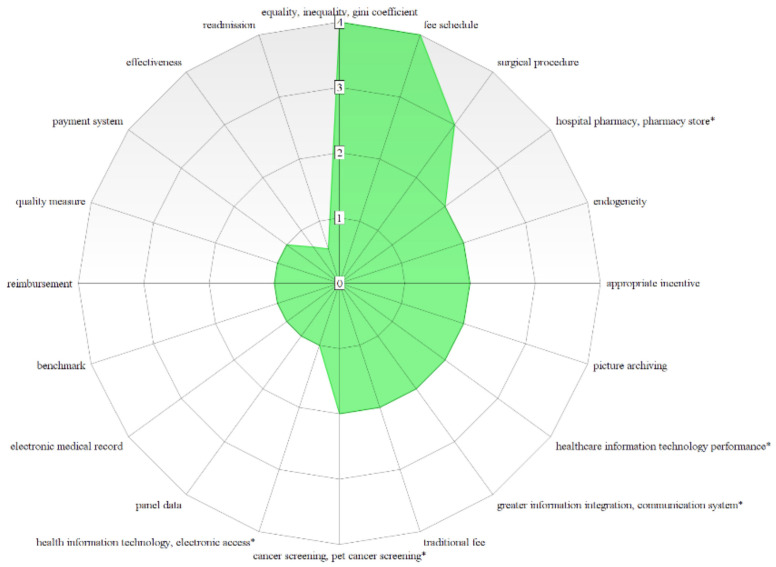
Hot Topics for a Research Agenda in the Green Cluster.

**Figure 11:**
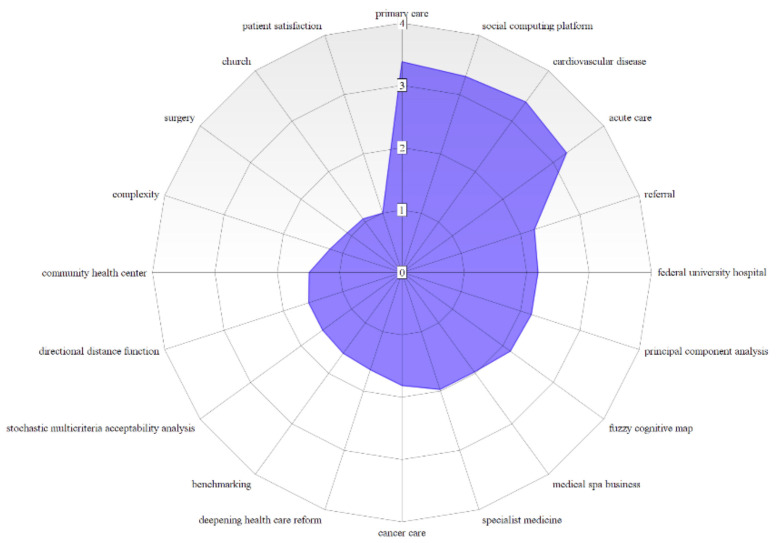
Hot Topics for a Research Agenda in the Blue Cluster.

About hospital sectors, activities and categories, applications related to church hospitals (0.941), cancer (0.500), surgical procedure (0.333), primary care (0.296) and pharmaceutical supplier (0.125) present the most notable topics; health care reform (0.609), economic development (0.500), inter-regional difference, equality, inequality, Gini coefficient (0.250), hazardous waste (0.500) and eco-efficiency (0.333) are the prominent concepts that can be explored with regard social, economic and environmental prospects. Concerning the class Information Technology, Systems and Communication, picture archiving and communication systems (0.500), and social computing platform (0.302) are the topics that are worth attention in future healthcare performance applications. Figure 9, Figure 10 and Figure 11 illustrate through radar charts the selected topics according to the relevance score for each topic on each cluster. The charts were created with the support of https://www.onlinecharttool.com/, accessed on 22 January 2022.

## 5. Conclusions

This work provided a comprehensive bibliometric investigation of the state-of-the-art frontier applications in hospitals using core publications, cluster analysis and co-occurrence networks. In the introduction, we proposed to address four research questions with this systematic bibliometric investigation. For the first question (*RQ1*)*: “Who are the most prominent contributions to the core literature of the DEA for the efficiency evaluation of hospitals?”* we report in the Systematic Review Design and in the Core Publications and Research Impact sessions the most recurrent contributors, the most relevant publications in the core of healthcare efficiency ranked by an attractiveness metric (citations over time), and two different bibliometric classifications, one for the core and the one for the broad set of publications. The clusters for the 66 core publications highlighted the research impact in the field with a comprehensive discussion on the 12 top-cited core publications and the most relevant contributions.

The research questions (*RQ2*): “What are the main quantity and quality variables used in the literature on hospital efficiency evaluation through DEA?” and (*RQ3*): “What are the main models and support approaches used in the literature on hospital efficiency evaluation through DEA? Which of these are most suitable for including different inputs and outputs combinations?” were addressed in the third and fourth sessions reporting the most common inputs, outputs and models, addressed by a timeline-based and distance-based network visualizations with a responsive discussion on the investigations and healthcare perspectives present in the most relevant core contributions. The traditional CCR and BCC are, without any doubt, the most recurrent used non-parametric approaches. In addition, Bootstrap DEA, Malmquist (MPI), Two-Stage DEA and some Network models have been given particular attention in the context of healthcare efficiency. Most of those models use staff, beds and different expenditure types as common resources to produce some interesting, even controversial outputs, such as emergency visits, ordinary and ICU admissions, consultations, intermediate products, and some undesirable outputs, such as in-hospital mortality, the length of hospitalization stays and readmissions.

Lastly, to address the research question (*RQ4*): “What are the main gaps and overlaps in the literature on hospital efficiency evaluation through DEA? How can we use this perspective to construct and address future research agendas in the field?” we construct the clusters for the 203 Web of Science papers in the Cluster Analysis session. The distance-based network visualizations were investigated based on the recurrent keywords and topics reported in the co-occurrence networks for outlining a research agenda. Selected hot topics for this research agenda were investigated based on degrees of generality that identify gaps and overlaps in the research field. The topics and discussions related to efficiency concepts, econometric support approaches, financial, environmental, social and economic prospects, hospital sectors, segments and technologies, among others, can offer an interesting managerial perspective for scholars, practitioners and analysts of healthcare efficiency. We hope this research can aid such prospects in the healthcare management industry.

## 6. Update

On 12 June 2022, at 11:43 am (UTC-03:00), we applied the final query string (Q4) for updating the systematic search with possible recent publications that were not covered in this investigation. This search results in the following additional references, which we kindly invite readers to consult [87,232,233,234,235,236,237,238,239,240,241,242,243,244,245,246].

## Figures and Tables

**Table 1:** Strings used in the Web of Science systematic search.

Acronym	Query String	Query Link
(Q1)	ALL = (healthcare AND efficiency)	[19]
(Q2)	“healthcare” AND efficiency (All Fields) and Articles (Document Types).	[20]
(Q3)	healthcare AND efficiency (All Fields) and “Data envelopment analysis” OR “Stochastic frontier analysis” (Topic) and Articles (Document Types).	[21]
(Q4)	healthcare AND efficiency (All Fields) and “Data envelopment analysis” OR “Stochastic frontier analysis” (Topic) and Hospital OR Hospitals (Topic) and Articles (Document Types)	[22]

**Table 2:** Core-publications clusters, variables and models.

Cluster	Common Inputs	Common Outputs	Models and Methods	Core Publications
Blue	Beds, bassinets, doctors (physicians), nurses, interns/residents, other medical staff, expenditures, hospital area (m^2^), doctor’s offices, healthcare institutions, assets, service complexity, inpatients, outpatients, unmet demand	Number of discharges, surgeries, infant deliveries, inpatient and outpatient treatments, income, diagnoses, operations, bed utilization, bed turnover, antenatal care, post-natal care, terminations of pregnancy (abortion), male and female sterilizations, admissions, case-mix adjusted admissions and discharges; outpatient visits (consultations), number of (FTE) trainees; In-hospital mortality rate, readmission, patient days (stays)	CCR (Constant Returns to Scale–CCR), BCC (Variable Returns to Scale–VRS), Bootstrap DEA (CRS and VRS), Malmquist (MPI), Two-Stage DEA, Non-oriented slack-based, Dynamic-Network DEA, Bootstrap Malmquist Productivity Index, Scale Efficiency.	[6,7,23,31,39,58,59,60,61,67,68,69,70,71,72,73,74,75,76,77,78,79,80,81,82,83,84,85,86,87,88,89,90,91,92,93,94]
Green	Beds, doctors (physicians), nurses, interns/residents, other medical staff, expenditures (medical staff costs, pharmacy and general costs), queue-related metrics (waiting to be seen, length of the queue, consultation time, service time at the pharmacy)	Number of discharges, surgeries, number of hospitalization days/bed-days, bed utilization, male patients treated, examinations in outpatient clinics, admissions and discharges, case-mix adjusted inpatient cases, outpatient visits (consultations), number of lab tests, number of pharmacists, number of emergency patients, number of referrals, patient days (stays)	CCR (Constant Returns to Scale–CCR), BCC (Variable Returns to Scale–VRS), Bootstrap DEA (CRS and VRS), Malmquist (MPI), Bootstrap Malmquist Productivity Index, Scale Efficiency, Categorical DEA, DEA-based Fuzzy and Two-stage Multicriteria.	[24,38,43,45,46,48,95,96,97,98,99,100,101,102,103,104,105,106,107,108,109,110,111,112,113,114,115,116]

**Table 3:** Most relevant contributions.

Reference	Title	Citation Score	Google Citations	Attractiveness
Kohl et al. (2019) [6]	The use of Data Envelopment Analysis (DEA) in healthcare with a focus on hospitals	16	182	3.96
Gok and Sezen (2013) [83]	Analyzing the ambiguous relationship between efficiency, quality and patient satisfaction in healthcare services: The case of public hospitals in Turkey	15	136	1.35
Cheng et al. (2015) [61]	Technical efficiency and productivity of Chinese county hospitals: an exploratory study in Henan province, China	15	87	1.14
Kawaguchi, Tone and Tsutsui (2014) [72]	Estimation of the efficiency of Japanese hospitals using a dynamic and network data envelopment analysis model	14	110	1.09
Mitropoulos, Talias and Mitropoulos (2015) [109]	Combining stochastic DEA with Bayesian analysis to obtain statistical properties of the efficiency scores: An application to Greek public hospitals	9	78	0.97
Valdmanis, Rosko and Mutter (2008) [23]	Hospital Quality, Efficiency, and Input Slack Differentials	12	152	0.96
Cheng et al. (2016) [60]	Efficiency and productivity measurement of rural township hospitals in China: a bootstrapping data envelopment analysis	9	58	0.93
Kounetas and Papathana-ssopoulos (2013) [107]	How efficient are Greek hospitals? A case study using a double bootstrap DEA approach	14	91	0.83
Caballer-Tarazona et al. (2010) [79]	A model to measure the efficiency of hospital performance	10	108	0.80
Jat and Sebastian (2013) [84]	Technical efficiency of public district hospitals in Madhya Pradesh, India: a data envelopment analysis	10	73	0.74
Lee, Chun and Lee (2008) [70]	Reforming the hospital service structure to improve efficiency: Urban hospital specialization	9	116	0.72
Lee, Yang and Choi (2009) [71]	The Association between Hospital Ownership and Technical Efficiency in a Managed Care Environment	9	95	0.59

**Table 4:** Cluster Definitions and Keywords.

Cluster	Definition	Publications
**Red**	Econometric Support Approaches, Environmental, Social and Economic Prospects in the Efficiency Analysis of Hospitals	[27,28,42,46,50,55,61,64,73,75,76,77,78,82,86,87,89,91,94,95,96,97,98,99,100,101,102,103,104,105,106,107,108,109,110,111,112,113,114,115,116,117,118,119,120,121,122,123,124,125,126,127,128,129,130,131,132,133,134,135,136,137,138,139,140,141,142,143,144,145,146,147,148]
**Green**	Financial and Managerial Perspectives, Information technologies in the Efficiency Analysis of Hospitals	[6,7,23,24,25,26,27,28,29,30,31,32,33,38,39,40,41,42,43,49,50,51,53,54,55,56,57,68,72,75,77,78,82,83,85,86,87,89,91,94,107,109,149,150,151,152,153,154,155,156,157,158,159,160,161,162,163,164,165,166,167,168,169,170,171,172,173,174,175,176,177,178,179,180,181,182,183,184,185,186,187,188,189,190,191,192,193,194,195,196,197,198,199,200,201,202]
**Blue**	Hospital Sectors or Activities and Healthcare Categories in the Efficiency Analysis of Hospitals	[34,35,36,37,38,39,46,47,48,51,58,60,61,67,69,71,73,80,88,90,92,93,95,103,104,108,112,114,116,120,161,203,204,205,206,207,208,209,210,211,212,213,214,215,216,217,218,219,220,221,222,223,224,225,226,227]
**Yellow**	Religious Perspectives in the Efficiency Analysis of Hospitals	[52]

**Table 5:** Recurrent Keywords reported in the Co-occurrence Networks.

Cluster	Most Recurrent Keywords	Publications
**Red**	data envelopment analysis, efficiency, hospitals, performance, technical efficiency, models, dea, data envelopment analysis (dea), healthcare, performance evaluation	[27,28,42,46,50,55,61,64,73,75,76,77,78,82,86,87,89,91,94,95,96,97,98,99,100,101,102,103,104,105,106,107,108,109,110,111,112,113,114,115,116,117,118,119,120,121,122,123,124,125,126,127,128,129,130,131,132,133,134,135,136,137,138,139,140,141,142,143,144,145,146,147,148]
**Green**	data envelopment analysis, efficiency, quality, hospitals, care, technical efficiency, impact, performance, productivity, bootstrap	[6,7,23,24,25,26,27,28,29,30,31,32,33,40,41,42,43,49,50,51,53,54,55,56,57,68,72,75,77,78,82,83,85,86,87,89,91,94,107,109,171,172,173,174,175,176,177,178,179,180,181,182,183,184,185,186,187,188,189,190,191,192,193,194,195,196,197,198,199,200,201,202]
**Blue**	data envelopment analysis, efficiency, technical efficiency, dea, health-care, quality, healthcare, hospitals, models, public hospitals	[34,35,36,37,38,39,46,47,48,51,58,60,61,67,69,71,73,80,88,90,92,93,95,103,104,108,112,114,116,120,161,203,204,205,206,207,208,209,210,211,212,213,214,215,216,217,218,219,220,221,222,223,224,225,226,227,228,229,230]
**Yellow**	religious diversity, health team, cultural, diversity, efficiency, data envelopment analysis	[52]

**Table 6:** Selected Topics and Degree of Generality for a Research Agenda.

Areas	Topics	Degree of Generality
Efficiency Concepts, Financial and Managerial Perspectives	integrated quality, undesirable output, uncertainty, complexity, benchmarking, patient satisfaction, fee schedule, traditional fee, reimbursement, quality measure, payment system, effectiveness, readmission.	From 2.178 (complexity) to 0.333 (integrated quality) in the red cluster. From 0.999 (patient satisfaction) to 0.624 (benchmarking) in the blue cluster. From 1.810 (readmission) to 0.250 (fee schedule) in the green cluster.
Concepts, Methods and Support Approaches	allocation efficiency, healthcare supply chain, integrated approach, ahp, fuzzy cognitive map, fuzzy data, tobit regression, tobit model, bootstrap, multiple stage approach, principal component analysis, stochastic multicriteria acceptability analysis, directional distance function, panel data	From 1.035 (multiple stage approach) to 0.250 (allocation efficiency) in the red cluster. From 0.635 (directional distance functions) to 0.458 (principal component analysis) in the blue cluster. One (1) in the green cluster (panel data).
Hospital Sectors and Healthcare Categories	pharmaceutical supplier, rural public health resource, voluntary agreement, rural medical service system, laboratory, public hospital, primary care, cardiovascular disease, acute care, referral, federal university hospital, medical spa business, specialist medicine, cancer care, community health center, surgery, church, surgical procedure, hospital pharmacy, pharmacy store, cancer screening, pet cancer screening.	From 2.027 (public hospital) to 0.125 (pharmaceutical supplier) in the red cluster. From 0.941 (church) to 0.296 (primary care) in the blue cluster. From 0.500 (cancer screening) to 0.333 (surgical procedure) in the green cluster.
Social and Economic Prospects	inter and intraregional difference, economic development, developed region, underdeveloped region, deepening health care reform, equality, inequality, gini coefficient.	From 0.500 (economic development) to 0.250 (inter regional difference) in the red cluster. 0.609 in the blue cluster (deepening health care reform). 0.250 in the green cluster (equality, inequality, gini coefficient).
Environment	eco-efficiency, environmental management, hazardous waste, waste, incinerator,	From 0.500 (hazardous waste) to 0.333 (eco efficiency) in the red cluster.
Information Technology, Systems and Communication	social computing platform, picture archiving, healthcare information technology performance, health information technology, electronic access, electronic medical record	0.302 in the blue cluster (social computing platform). From 1 (electronic medical record) to 0.500 (picture archiving) in the green cluster.
Miscellaneous	geographic elevation, higher altitude, altitude, sea level, endogeneity, appropriate incentive.	0.333 in the red cluster (geographic elevation, higher altitude, altitude, sea level), 0.500 (endogeneity and appropriate incentive) in the green cluster.

## Data Availability

Not applicable.
